# Patterns and Predictors of Abortion Care-Seeking Practices in India: Evidence From a Nationally Representative Cross-Sectional Survey (2019-2021)

**DOI:** 10.7759/cureus.41263

**Published:** 2023-07-01

**Authors:** Mansi Malik, Siaa Girotra, Mrunali Zode, Saurav Basu

**Affiliations:** 1 Indian Institute of Public Health-Delhi, Public Health Foundation of India, New Delhi, IND

**Keywords:** health services, nfhs-5, abortion, maternal mortality, maternal health, reproductive health

## Abstract

Background

India continues to have unsafe abortions despite progressive legislation since the past five decades facilitating ease of access to abortion services. This study describes abortion care-seeking patterns (social/therapeutic/humanitarian/sex-selective/safe/unsafe), preferences (public/private/at home), and their determinants among Indian women.

Methods

Data were taken from the Indian National Family and Health Survey (NFHS-5) (2019-2021) including women aged 15-49 years, who had terminated their last pregnancy by induced abortion within five years prior to the survey (N = 5,856). A bivariate analysis, followed by a multinomial logistic regression model, was performed to assess the predictors affecting the choice of healthcare facility type for an abortion. Predictors of unsafe and self-managed abortions were examined using binary logistic regression.

Results

About 665,671 women in the reproductive age group responded to the survey, of which 3.42% (n=22,767) reported their most recent pregnancy within the last five years terminated in either a miscarriage, stillbirth or abortion, of which 5,856 (25.72%) underwent an induced abortion. Women undergoing surgical abortion were more likely to avail of either a public (adjusted relative risk ratio (aRRR)=38.06 (23.62, 61.35)) or a private facility (aRRR=44.53 (28.11,70.53)) compared to at-home abortions. Women reporting a social and humanitarian reason for abortion were less likely to undergo an abortion at a public (aRRR=0.25 (0.17,0.35)) or private facility (aRRR=0.32 (0.23,0.44)) than at home. Furthermore, a total of 147 (2.43%) abortions were classified as unsafe. Women reporting sex-selective reasons for abortion were observed to have a higher likelihood of engaging in an unsafe abortion (adjusted odds ratio (aOR)= 1.61 (0.70, 3.70)) compared to those citing a therapeutic reason.

Conclusions

Self-managed abortions at home were more prevalent in women of lower socioeconomic status, adolescent girls, and those reporting sex-selective reasons for abortion. Furthermore, the reproductive-health program in India should enhance capacity-building initiatives for primary-care healthcare providers, including doctors, nurses, and pharmacists, to effectively prescribe and supervise abortion through medication methods.

## Introduction

Abortion is a healthcare intervention and is regarded as a safe medical procedure when performed in accordance with a method recommended by the World Health Organization (WHO) that is appropriate for the gestational age and by trained medical professionals [1]. Induced abortion is defined as "pregnancy terminated voluntarily from a service provider" [2]. A medical abortion can also be effectively and safely self-managed by pregnant women outside of a healthcare facility (such as at home) during early pregnancy [1].

The WHO defines unsafe abortions as "the termination of an unintended pregnancy by persons lacking the essential skills or in an environment lacking the minimum medical standards, or both" [3]. According to global estimates, nearly half (45%) of the 73 million abortions performed worldwide each year are unsafe, with 97% of these unsafe abortions occurring in low- and middle-income countries (LMICs) [4,5]. Unsafe abortions are attributed to causing a high burden of potentially life-threatening complications, such as hemorrhage, infection, and trauma with residual morbidity from chronic health conditions, and often irreversible physical and mental health problems leading to long-term risk of anxiety, depression, and post-traumatic stress disorders [6,7].

Abortions have been fully legal in India under various circumstances since the enactment of the Medical Termination of Pregnancy (MTP) Act in the 1970s [8]. The MTP Act enables registered qualified medical practitioners at certified facilities to provide abortion services to save a woman’s life; to preserve her mental or physical health; in case of an economic or a social necessity; in case of rape, incest, or fetal impairment; and, in the event of a contraceptive failure. The act was amended in 2002-3 to devolve the approval process for a private facility to provide abortion services from the state level to the district-level committee to expand the number of providers offering comprehensive abortion care services within the legal framework. In 2018, the Government of India further issued guidelines to train doctors at public-health facilities on comprehensive abortion care, including both medication and surgical abortion [9]. The MTP Act has been recently amended in 2021 again, facilitating further ease of access to legal and safe abortion services for all women regardless of their marital status. Until recently, conducting an abortion needed one medical opinion if it was performed within the first 12 weeks of conception and two medical opinions if it was performed between 12 and 20 weeks. However, as per the modified MTP Act, 2021, all pregnant women can now terminate a pregnancy up to 20 weeks of gestation on the advice of only one medical professional, and women who are survivors of sexual abuse, victims of rape or incest, or disabled can seek termination up to 24 weeks [10].

Unfortunately, a large proportion of women in India continue to utilize illegal and potentially unsafe abortions that jeopardize their health and contribute to significant mortality [11]. It is estimated that in India 77% of unintended pregnancies end in abortion, while only 22% of abortions were considered safe [12,13]. Approximately, 8% of maternal mortality was attributed to unsafe abortions in 2018, and nearly eight women die each day due to causes related to unsafe abortion in the country [14,15].

Prior research has shown that living in rural regions, not having children, having less education, being exposed to the media, poor antenatal care (ANC) utilization, younger age of mothers, and maternal nutritional status were all factors that were significantly associated with a high risk of undergoing unsafe abortion [16,17]. Moreover, the high burden of unsafe abortion can also be attributed to poverty, social inequity, and denial of women’s human rights [18].

According to data from India's National Family Health Survey (NHFS)-fourth round (2015-2016), married women who experienced intimate partner violence were more likely to have abortions through self-management [19]. Additionally, women, particularly adolescent girls and those who are poor and/or living in rural areas, often lack information about the legal status of abortions in their country and where to seek safe abortion services. They may also frequently lack the decision-making power and financial resources to seek such services, or they might be discouraged by healthcare providers’ negative attitudes and a lack of confidentiality and privacy. Moreover, the stigma associated with abortions, especially in unmarried women, may prevent women from accessing safe abortion services. Healthcare providers who offer these services may perceive discrimination causing them to be reluctant in providing these services [20]. These conflicts may cause moral distress and undermine the doctor-patient relationship [18,21].

The Sustainable Development Goals (SDGs) for 2030 have renewed governments’ commitments, made under the Millennium Development Goals (MDGs), to focus on ensuring universal access to sexual and reproductive health (SRH) services. Availability and access to safe abortion services are integral components of SRH services and are needed to fulfill the SDG mandate of “leaving no one behind”. Therefore, advancing women’s access to safe and legal abortion is an urgent priority in accordance with the new SDGs focused on health and gender equality [22].

In India, in recent years, progressive legislation affirmed by judicial decisions has considerably advanced access and availability to universal and comprehensive abortion care services while protecting women's autonomy and confidentiality [9,10]. Consequently, the impact of such legislation on real-world access to abortion care services and the burden of unsafe abortions warrants further exploration. Understanding the determinants of women’s choices in selecting abortion services is essential for safeguarding their health during this socially and medically vulnerable situation that may have lifetime health consequences [23].

Studies with small sample sizes from non-representative geographic data constitute a majority of the evidence on abortion care in India. The proposed study will contribute towards an improved understanding of the change in patterns of abortion care-seeking practices and their determinants based on the updated round of India's demographic and health survey data. The study’s objectives were to describe abortion care-seeking patterns (social/ therapeutic/humanitarian/sex-selective/safe/unsafe), preferences (public/private/home), and their determinants among women in India. We further explored the predictors associated with unsafe abortion practices.

## Materials and methods

Data source

The study analyses data from the fifth series of India's National Family and Health Survey (2019-21) (NFHS-5). Data on India's population and health are available for 707 districts, 28 states, and eight union territories from 636,699 households. NFHS-5 is a two-stage stratified sample. Villages in rural areas and census enumeration blocks (CEBs) in urban areas served as the primary sample units (PSUs), and these PSUs were chosen using the probability proportional to size (PPS) sampling method [24]. A total of 724,115 eligible women aged 15-49 years were interviewed using standardized questionnaires in all states of the country. The analysis of this study included data from women aged 15 to 49 years of any marital status who had terminated their last pregnancy by induced abortion (and not spontaneous) in the five years preceding the survey. The five years between the start and end of the fieldwork period were considered, which included a total of 5,856 women with a self-reported history of induced abortion.

Inclusion Criteria 

The analysis included face-to-face interview data from women aged 15 to 49 years of any marital status who had terminated their last pregnancy by induced abortion, i.e., the deliberate interruption of an ongoing pregnancy by medical or surgical means (not spontaneous abortion, i.e., a miscarriage) in the five years preceding the survey.

Outcome variable

The primary outcome variable was “Place the last termination was performed”, which was broadly classified into the three categories of public facility, private facility, and at home.

Public facilities included: public: govt. / municipal hospital, public Ayurveda, Unani, Yoga, Siddha, Homeopathy (AYUSH): Ayurveda, public AYUSH: yoga and naturopathy, public AYUSH: Unani, public AYUSH: Siddha, public AYUSH: homeopathy, public AYUSH: public AYUSH: other, public: govt. dispensary/clinic, public: UHC / UHP / UFWC, public: CHC / rural hospital/block PH, public: PHC / additional PHC, public: sub-center, public: govt. the mobile clinic, and other public health. Private facilities included: Non-Government Organizations (NGO) or trust hospitals/clinics, private: hospitals/clinics, private: dispensaries/clinics, and other private health sectors. 

The home category consisted of only at-home abortions. The secondary outcome variable was unsafe abortion. Abortions were considered unsafe if they met any of the following criteria: a) First trimester, place of abortion: home, method of abortion: surgical; b) Second trimester, place of abortion: home, method of abortion: medication; c) Second trimester, method of abortion: surgical, conducted by self/non-skilled attendant; d) Third trimester, beyond >24 weeks of gestation.

Independent variables

Variables that were known to influence the decision-making in seeking different health facility alternatives for abortion care from previous literature were examined for their association with the outcome variable. Individual level determinants included factors such as woman’s age, education, and marital status; household factors such as religion, caste, household members, and wealth index; community-level characteristics such as place of residence, and region; pregnancy and abortion characteristics such as parity, the reason for abortion, etc.

Region Variable

The region was classified based on the state variable such as North (Jammu & Kashmir, Himachal Pradesh, Punjab, Chandigarh, Uttarakhand, Haryana, NCT of Delhi, Rajasthan, and Ladakh); Central (Uttar Pradesh, Madhya Pradesh, and Chhattisgarh); East (Bihar, West Bengal, Jharkhand, and Odisha); Northeast (Sikkim, Arunachal Pradesh, Nagaland, Manipur, Mizoram, Tripura, Meghalaya, and Assam); West (Gujarat, Dadra & Nagar Haveli, Daman & Diu, Maharashtra, and Goa); South (Andhra Pradesh, Karnataka, Lakshadweep, Kerala, Tamil Nadu, Puducherry, Andaman & Nicobar Islands, and Telangana). 

Caste Variable

Community was classified into Scheduled Caste (SC), Scheduled Tribe (ST), Other Backward Castes (OBCs), and other. The SC and ST communities are officially considered the most socio-economically disadvantaged groups in India [25]. 

Marital Status Variable

It was integrated into three categories: unmarried, married, and divorced/separated/widowed. 

Composite Independent Variables 

Exposure to mass media: Females who either read newspapers or magazines or listen to the radio or watch television were considered to have some mass media exposure compared to those who did not fall into any of the above scenarios.

Comprehensive women empowerment: In this study, a woman was considered empowered if she said yes to all the following: Owned a mobile phone, had a bank/savings account, was at least educated up to high school, and could take decisions when it comes to money. In previous studies, factors such as a woman's ability to make decisions involving care of herself, employment status, making a major household purchase, visits to her family and relatives, owning a house/land alone or jointly with her husband, and using hygienic methods of protection during her menstrual period are also considered when focusing on comprehensive women empowerment [26]. However, owing to a lack of data points these factors were not considered in our analysis.

Domestic Violence

In the NFHS-5 survey, only 72,056 women completed the domestic violence module [27]. Furthermore, in our study sample (n=5856), data points were available for only 12.89% (n= 755) of women for the domestic violence module. Women who responded ‘yes’ to any of the following questions were categorized as having experienced domestic violence: Experienced any less severe violence by husband/partner; Experienced any severe violence by husband/partner; Experienced any sexual violence by husband/partner; Previous husband: ever hit, slap, kick or physically hurt respondent; Previous husband: physically forced to have sex or to perform sexual acts; Person other than husband/partner ever physically hurt respondent; Respondent was hurt by anyone during a pregnancy; Ever forced to perform unwanted sexual acts.

Trimester Variable

Trimester at which abortion was performed was categorized into first trimester (0-3 months), second trimester (4-6 months), and third trimester (seven months and above).

Reason for Abortion Variable

The main reason for abortion as reported by the respondent were grouped as follows: Therapeutic (Health did not permit or complications in pregnancy); Humanitarian and Social (Humanitarian: Fetus had a congenital abnormality or contraceptive failure; Social:Economic reasons, last child too young, unplanned pregnancy, husband/mother-in-law did not want) and Sex-selective (male fetus, female fetus).

Methods of Abortion

The methods were categorized as surgical and medical abortions.

Statistical analysis

All the data points were checked for their plausibility before initiating the analysis. The sample included women of the 15-49 age group who reported having their last pregnancy terminated in an induced abortion preceding five years from the survey. The respondents with missing values in outcome variable were excluded and the final sample size, therefore, was 5,856 women irrespective of their marital status. Independent variables were first described extensively after setting up the data for survey analysis. We performed analysis using Stata’s svyset command to account for sampling weights, clustering, and stratification. All the weighted percentages along with the frequencies segregated by type of health facility were reported for each exposure variable. Additionally, observations were considered as missing and excluded from the denominator for ‘don’t know’ responses.

Since the outcome variable had more than two unordered categories, multinominal logistics regression (MNLR) was performed to calculate the relative risk ratio (RRR) at a 5% significance level. In the MNLR model, at-home was kept as the reference category, and RRR were calculated for public and private healthcare facilities. Variables with p-value <0.05 of the crude model were carried forward in the adjusted model. Adjusted MNLR was performed to evaluate the independent effect of each factor variable on the outcome after adjusting for other variables. These results are represented as aRRR with their 95% confidence intervals (CI). All the model assumptions such as linearity of logit (log odds of outcome), absence of outliers, and independence of irrelevant alternatives (IIA) were checked. No issue of multicollinearity was observed in the data. Multicollinearity was assessed for continuous factors where a correlation of >0.8 was considered to be multicollinear. Furthermore, binary logistic regression was performed to assess predictors of self-managed abortions and unsafe abortions. The binary logistic model fit was assessed using the Hosmer-Lemeshow test. These results are represented as adjusted odds ratio (aOR) with their 95% CI. All the analysis was performed in STATA version 15.1 (Stata Corporation, College Station, USA).

Ethics statement

This study is the secondary data analysis of de-identified data from the publicly accessible NFHS-5 dataset for India. The original survey participants voluntarily provided signed informed consent, and the survey protocol received approval from the institutional review board at the International Institute for Population Sciences (IIPS), Mumbai. After reviewing the submitted proposal by the authors, Demographic Health Survey (DHS) granted access to the dataset. None of the authors had access to the information that could identify individual participants during or after data collection.

## Results

In the survey, 665,671 women in the reproductive age group were interviewed, of which 3.63% (n=22,767) reported that their last pregnancy within the previous five years terminated in a miscarriage, stillbirth, or abortion. Among this 3.63% of women, 5,856 (26.21%) reported having undergone an induced abortion. However, a total of 5,824 women undergoing induced abortion gave information for the site at which abortion was obtained. The proportion of women in India who underwent an induced abortion at home, public health facilities, and private health facilities was 25.90% (n=1612), 19.44% (n=1418), and 54.66% (n= 2794), respectively.

Characteristics of the women who had terminated their last pregnancy by abortion in the last five years, stratified by the type of health facility attended for the abortion, are reported in Table 1. A higher proportion of women across all age groups regardless of their marital status, education level, place, and region of residence availed of private facilities for abortion-care services whereas a greater proportion of women with husbands lacking primary or without formal education underwent abortion at home.

**Table 1:** Background characteristics of women aged 15-49 whose last pregnancy ended in an induced abortion stratified by place of abortion (N=5,824), NFHS-5 (2019-20) NHFS: National Family and Health Survey; SC: Scheduled Caste; ST: Scheduled Tribe; OBC: Other Backward Caste

Characteristic	Public (row %) N= 1,418	Private (row %) N= 2794	Home (row %) N= 1,612	Total n (col%)
Individual level				
Age (in years) (n=5,824)				
≤19	27 (15.87)	78 (61.73)	42 (22.40)	147 (2.55)
20-29	771 (18.26)	1,608 (54.22)	1,008 (27.51)	3,387 (59.30)
30-39	548 (2 6)	995 (55.13)	504 (23.21)	2,047 (34.48)
≥40	72 (20.09)	113 (52.41)	58 (27.51)	243 (3.66)
Marital status				
Unmarried	8 (6.27)	35 (67.92)	34 (25.81)	77 (0.65)
Married	1,393 (19.48)	2729 (54.58)	1,562 (25.95)	5,684 (98.29)
Separated/widowed	17 (24.06)	30 (54.32)	16 (21.62)	63 (1.06)
Education attainment				
No education	193 (19.04)	334 (48.10)	249 (32.86)	776 (13.25)
Primary	199 (23.87)	250 (42.96)	206 (33.17)	655 (10.78)
Secondary	847 (20.74)	1,536 (52.94)	958 (26.32)	3,341 (55.52)
Higher	179 (13.83)	674 (69.76)	199 (16.42)	1,052 (20.44)
Husband's education				
No education	26 (29.64)	21 (28.41)	27 (41.95)	74 (7.75)
Primary	24 (19.03)	30 (37.78)	43 (43.19)	97 (9.98)
Secondary	135 (17.89)	235 54.21	176 (27.90)	546 (62.28)
Higher	35 (15.78)	107 (64.75)	39 (19.48)	1,81 (19.98)
Comprehensive women empowerment (n=911)				
No	158 (19.43)	269 (49.04)	210 (31.53)	637 (69.72)
Yes	63 (15.89)	129 (60.70)	82 (23.40)	274 (30.28)
Exposure to mass media				
No exposure	297 (22.85)	409 (44.89)	363 (32.26)	1,069 (17.93)
Any exposure	1,121 (18.69)	2,385 (56.80)	1,249 (24.51)	4,755 (82.07)
Domestic violence (N=753)				
No	107 (17.19)	206 (54.42)	141 (28.39)	454 (63.14)
Yes	74 (17.23)	121 (47.95)	104 (34.82)	299 (36.86)
Correct knowledge of ovulatory cycle				
No	929 (19.04)	1,937 (54.43)	1,155 (26.53)	4,021 (70.88)
Yes	489 (20.42)	857 (55.22)	457 (24.36)	1,803 (7.09)
Distance to health facility a problem				
No problem	596 (18.67)	1,311 (55.88)	683 (25.46)	2,590 (47.67)
Big problem	348 (19.08)	641 (54.42)	401 (26.49)	1,391 (21.38)
Not a big problem	473 (20.88)	842 (52.96)	528 (26.17)	1,843 (30.95)
Getting money for treatment				
No problem	648 (17.81)	1,562 (57.92)	773 (24.28)	2,983 (53.48)
Big problem	355 (23.78)	455 (49.45)	320 (26.76)	1,130 (17.81)
Not a big problem	415 (19.79)	777 (51.83)	519 (28.38)	1,711 (28.70)
Trimester				
First	808 (16.51)	1,660 (49.87)	1,409 (33.62)	3,877 (67.42)
Second	543 (24.52)	1,023 (64.88)	197 (10.60)	1,763 (29.39)
Third	67 (34.43)	111 (61.91)	6 (3.66)	1,84 (3.19)
Parity				
0	132 (21.70)	336 (66.49)	95 (11.81)	563 (8.94)
1	430 (20.02)	864 (58.33)	416 (21.66)	1,710 (30.76)
≥2	856 (18.81)	1,594 (51.04)	1,101 (30.15)	3,551 (60.30)
Household level				
Religion				
Hindu	1,024 (18.26)	2,325 (54.90)	1,339 (26.84)	4,688 (85.23)
Muslim	219 (26.73)	250 (51.06)	145 (22.21)	614 (10.62)
Other	173 (24.66)	218 (59.18)	128 (16.17)	519 (4.15)
Caste (N=5,507)				
SC	307 (21.60)	560 (51.39)	366 (27.02)	1,233 (24.80)
ST	256 (27.60)	225 (42.81)	221 (29.59)	702 (6.32)
OBC	468 (17.76)	1,236 (56.36)	625 (25.88)	2,329 (44.88)
Non-SC/ST/OBC	265 (15.96)	662 (59.71)	316 (24.33)	1,243 (24.00)
Household size				
≤5 members	876 (20.71)	1,599 (54.94)	886 (24.34)	3,361 (57.47)
5+ members	542 (17.72)	1,195 (54.28)	726 (28.0)	2,463 (42.53)
Wealth quintile				
Poorest	282 (23.78)	299 (39.98)	351 (36.23)	932 (13.73)
Poor	357 (24.38)	468 (44.20)	407 (31.42)	1,232 (18.23)
Middle	331 (21.36)	628 (56.05)	314 (22.59)	1,273 (21.54)
Richer	270 (18.56)	669 (57.55)	297 (23.89)	1,236 (23.46)
Richest	178 (12.05)	730 (67.45)	243 (20.50)	1,151 (23.04)
Community level				
Place of residence				
Urban	354 (17.57)	926 (60.31)	401 (22.12)	1,681 (37.07)
Rural	1,064 (20.54)	1,868 (51.33)	1,211 (28.12)	4,143 (62.93)
Region				
North	296 (22.21)	484 (48.24)	271 (29.56)	1,051 (12.39)
Central	219 (13.74)	677 (51.56)	487 (34.71)	1,383 (28.01)
East	194 (18.23)	430 (43.99)	444 (37.78)	1,068 (22.14)
Northeast	406 (44.13)	265 (27.95)	297 (27.91)	968 (5.50)
West	84 (14.64)	288 (75.94)	46 (9.42)	418 (12.55)
South	219 (23.39)	650 (69.22)	67 (7.39)	936 (19.42)

Table 2 reports the abortion-related characteristics among women aged 15-49 whose last pregnancy ended in an induced abortion. The most common reasons for having an abortion were reported as social causes (64.63%) such as unplanned pregnancy, husband/mother-in-law did not want, economic reasons, etc. Nearly 70% of the women stated they had undergone a surgical abortion and 80% of the women reported no complications following an abortion. However, only 10% of the women who had post-abortion complications sought medical care or treatment.

**Table 2:** Characteristics of abortion among women aged 15-49 who reported their last pregnancy ended in an induced abortion, NFHS-5 (2019-21) NFHS: National Family and Health Survey

Characteristics of Abortion	N=5856	Column %
Reason for getting an abortion (N=5,548)		
Therapeutic	1,364	24.79
Humanitarian	456	8.21
Social	3,599	64.63
Sex-selective	129	2.37
Method of abortion		
Surgical	3,960	68.94
Medicine	1,690	27.16
Any other	206	3.91
Site of abortion (N=5,824)		
Public	1,418	19.44
Private	2,794	54.66
At home	1,612	25.90
Complications from abortion (N=5,856)		
No	5,008	85.28
Yes	848	14.72
Sought treatment for complication (N=848)		
No	120	10.33
Yes	728	89.67
Treatment site for complications (N= 724)		
Public	251	26.57
Private	466	72.24
At home	7	1.20

**Figure 1:**
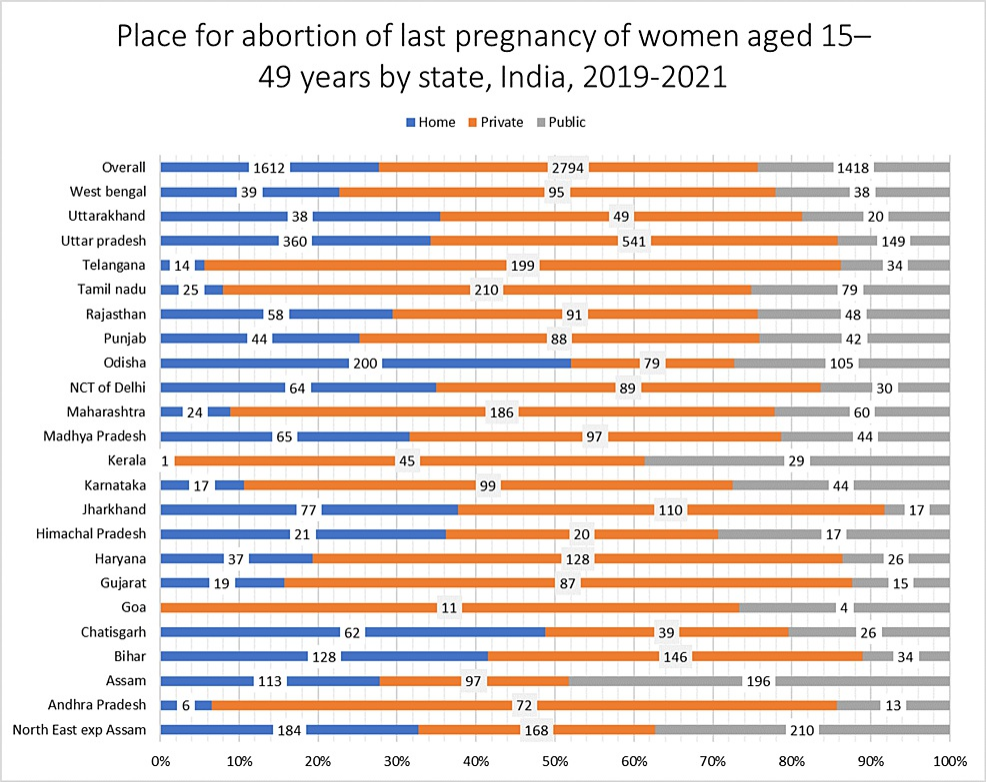
Place for abortion of last terminated pregnancy of women aged 15-49 years by state

In most of the states, private health facilities were utilized for abortion care services compared to public health facilities or at-home except for the states of Odisha, Chhattisgarh, and Bihar, where at-home abortions seemed more prevalent (Figure 1). Maximum utilization of public health facilities was observed in the states of Assam, Kerala, and Northeastern states (Figure 1). The results of the crude analysis are depicted in the supplementary material (Table 5). 

Table 3 presents the findings of an adjusted multinomial regression analysis that evaluated factors influencing the type of healthcare facilities accessed for abortion care. After adjusting for other predictors, women aged more than 30 years were approximately four times more likely to seek abortion care services at public facilities than undergo an abortion at home (aRRR= 4.73 (1.84,12.17) for 30-39 aged; aRRR= 4.19 (1.45,12.10) for ≥40 years). Also, women with higher educational levels were more likely to utilize abortion care services at public and private facilities compared to at home [(aRRR= 1.56 (0.94, 2.57) for public facility, aRRR= 1.49 (0.98, 2.26) for private facility)]. Women from the southern (aRRR=5.92 (3.64, 9.63) for public, aRRR= 10.24 (6.55, 16.03) for private) and western parts (aRRR=2.77 (1.58,4.86) for public, aRRR= 6.19 (3.69,10.38) for private) of India were more likely to undergo abortion in a health facility rather than at home. On the contrary, women from rural areas (aRRR= 0.84 (0.62, 1.13) for the public facility; aRRR= 0.88 (0.68,1.14) for the private facility) and reporting non-therapeutic reasons for abortion were less likely to undergo an abortion at either a public or private health facility.

**Table 3:** Factors associated with the place of abortion (public, private, home) for abortion care utilization in women aged 15-49 years (N=5,049) aRRR: Adjusted relative risk ratio; SC: Scheduled Caste; ST: Scheduled Tribe; OBC: Other Backward Caste *Testparm p-value

Variables	Public Facility		Private Facility		
	aRRR	P-value	aRRR	P-value	Overall p-value*
Individual level					
Age (in years)					
≤19	Ref		Ref		
20-29	2.17 (0.87,5.43)	0.098	1.34 (0.65,2.78)	0.426	
30-39	4.73 (1.84,12.17)	0.001	2.06 (0.97,4.38)	0.060	
≥40	4.19 (1.45,12.10)	0.008	1.95 (0.82,4.65)	0.133	<0.001
Education attainment					
No education	Ref		Ref		
Primary	1.51 (0.97, 2.33)	0.063	0.94 (0.64,1.38)	0.753	
Secondary	1.90 (1.31, 2.76)	0.001	1.28 (0.93,1.77)	0.132	
Higher	1.56 (0.94, 2.57)	0.081	1.49 (0.98,2.26)	0.063	0.002
Exposure to mass media					
No exposure	Ref		Ref		
Any exposure	0.94 (0.69, 1.28)	0.732	0.97 (0.73,1.28)	0.804	0.92
Getting money for treatment					
No problem	Ref		Ref		
Problem	1.38 (1.01, 1.90)	0.044	1.20 (0.90,1.60)	0.208	
Household level	1.10 (0.83, 1.45)	0.489	1.09 (0.86,1.38)	0.490	0.37
Religion					
Hindu	Ref		Ref		
Muslim	2.13 (1.38, 3.29)	0.001	1.33 (0.90,1.96)	0.152	
Other	1.81 (1.11, 2.94)	0.017	1.84 (1.21,2.80)	0.005	<0.001
Caste					
SC	1.65 (1.14, 2.37)	0.007	1.06 (0.77, 1.45)	0.712	
ST	1.45 (0.92, 2.28)	0.104	0.83 (0.54, 1.29)	0.425	
OBC	1.14 (0.82, 1.58)	0.421	0.96 (0.73, 1.27)	0.821	0.019
Non-SC/ST/OBC	Ref				
Household size					
<5 members	Ref		Ref		
5+ members	1.09 (0.85, 1.41)	0.424	0.96 (0.78, 1.18)	0.725	0.717
Wealth quintile					
Poorest	Ref		Ref		
Poor	0.90 (0.60, 1.31)	0.60	0.96 (0.68,1.34)	0.799	
Middle	0.94 (0.63, 1.39)	0.75	1.37 (0.95,1.95)	0.087	
Richer	0.56 (0.35, 0.88)	0.01	0.90 (0.61,1.33)	0.593	
Richest	0.41 (0.24, 0.69)	0.001	1.17 (0.75,1.84)	0.483	<0.001
Community level					
Place of residence					
Urban	Ref		Ref		
Rural	0.84 (0.62, 1.13)	0.26	0.88 (0.68,1.14)	0.323	0.573
Region					
North	Ref		Ref		
Central	0.57 (0.39, 0.83)	0.003	1.19 (0.87,1.63)	0.281	
East	0.67 (0.45, 0.98)	0.044	1.27 (0.90,1.80)	0.178	
Northeast	1.08 (0.69, 1.68)	0.72	0.50 (0.32,0.78)	0.002	
West	2.77 (1.58, 4.86)	<0.001	6.19 (3.69,10.38)	<0.001	
South	5.92 (3.64, 9.63)	<0.001	10.24 (6.55,16.03)	<0.001	<0.001
Pregnancy characteristic					
Parity					
0	Ref		Ref		
1	0.63 (0.36, 1.09)	0.10	0.63 (0.37,1.07)	0.087	
>2	0.49 (0.28, 0.85)	0.01	0.57 (0.34,0.97)	0.037	0.128
Abortion characteristics					
Reason for getting abortion					
Therapeutic	Ref		Ref		
Humanitarian and social	0.25 (0.18, 0.35)	<0.001	0.32 (0.23,0.45)	<0.001	
Sex selective	0.24 (0.09, 0.61)	0.003	0.51 (0.22,1.15)	0.103	<0.001
Method of abortion					
Medication	Ref		Ref		
Surgical	38.06 (23.62, 61.35)	<0.001	44.53 (28.11,70.53)	<0.001	<0.001

**Figure 2:**
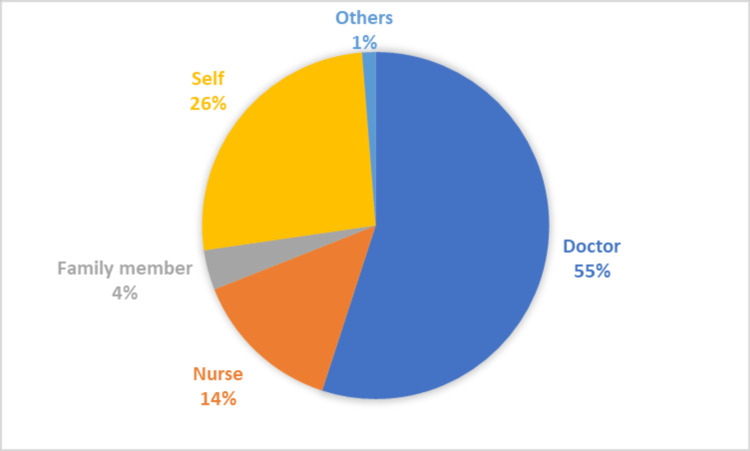
Distribution of the percentage of skilled and unskilled professionals who performed abortions

A majority (54.99%, n=3,123) of the abortions were performed by a medical doctor whereas 14.01% (n=838) were done by nursing practitioners. About 26.04% (n=1609) of abortions were self-managed and not supervised by any medical healthcare professional (Figure 2).

Of the total at-home abortions (n=1,612, 1.59% (n=31)) were surgical abortions. Amongst these surgical at-home abortions, 1.4% were reportedly performed by untrained individuals (family members (n=8) and self (n=13)) whereas 0.2% (n=6) were performed by trained doctors and nurses.

Predictors of self-managed abortions i.e., abortions performed at home during the first trimester using medication by the woman herself with or without supervision by a healthcare professional are reported in Table 4. A total of 1,501 (23.94%) women underwent an abortion during the first trimester using medication at home. On adjusted analysis, older women (≥40 years) (aOR=0.50 (0.23, 1.07)) compared to adolescents (≤19), women belonging to southern (aOR= 0.16 (0.11,0.25)), western (aOR= 0.26 (0.17,0.41)) or northeastern (aOR= 0.79 (0.57,1.09)) region compared to northern parts were less likely to undergo a self-managed abortion. However, self-managed abortions were more common among multiparous women (aOR= 1.87 (1.20, 2.92)). Women who reported humanitarian or social reasons for abortion had four times higher odds of self-managing an abortion compared to those who cited a therapeutic reason (aOR=4.50 (3.34, 6.07)).

**Table 4:** Predictors of women undergoing abortions at home in the first trimester with medication (self-managed abortions) NFHS-5 (2019-21) OR: Odds ratio; aOR: Adjusted odds ratio; CI: Confidence interval; NHFS: National Family and Health Survey estat gof p-value= 0.749 No. of observations in the model= 5,545

Variables	N (%) (n=1,501)	OR (95% CI)	p-value	aOR (95% CI)	p-value
Individual level					
Age (in years)					
≤19	38 (2.02)	Ref		Ref	
20-29	943 (63.73)	1.48 (0.89, 2.46)		0.79 (0.42,1.49)	
30-39	470 (30.51)	1.16 (0.69, 1.94)		0.49 (0.25,0.95)	
≥40	50 (3.74)	1.39 (0.75, 2.60)	0.02	0.50 (0.23,1.07)	<0.001
Education attainment					
No education	222 (16.22)	Ref		Ref	
Primary	193 (14.04)	1.09 (0.82, 1.45)		1.11 (0.81,1.51)	
Secondary	895 (56.56)	0.77 (0.62, 0.96)		0.92 (0.71,1.20)	
Higher	191 (13.18)	0.44 (0.33, 0.59)	<0.001	0.80 (0.56,1.15)	0.33
Exposure to mass media					
No exposure	326 (21.96)	Ref		Ref	
Any exposure	1,175 (78.04)	0.71 (0.58, 0.86)	<0.001	0.97 (0.77,1.22)	0.81
Household level					
Religion					
Hindu	1,246 (88.53)	Ref		Ref	
Muslim	135 (9.32)	0.80 (0.62, 1.05)		0.76 (0.57,1.03)	
Other	120 (2.15)	0.43 (0.31, 0.59)	<0.001	0.61 (0.43,0.87)	0.008
Wealth quintile					
Poorest	311 (18.52)	Ref		Ref	
Poorer	383 (22.30)	0.86 (0.68, 1.09)		1.03 (0.79,1.35)	
Middle	296 (19.13)	0.57 (0.44, 0.73)		0.93 (0.69,1.24)	
Richer	280 (21.34)	0.58 (0.45, 0.75)		1.16 (0.84,1.61)	
Richest	231 (18.70)	0.51 (0.39, 0.67)	<0.001	0.96 (0.66,1.41)	0.52
Community level					
Place of residence					
Urban	380 (31.62)	Ref		Ref	
Rural	1,121 (68.38)	1.36 (1.14, 1.63)	<0.001	1.05 (0.84,1.31)	0.63
Region					
North	254 (14.19)	Ref		Ref	
Central	447 (38.13)	1.27 (1.02, 1.59)		1.03 (0.80,1.33)	
East	416 (32.44)	1.40 (1.11, 1.78)		1.02 (0.77,1.35)	
Northeast	291 (6.26)	0.98 (0.76, 1.29)		0.79 (0.57,1.09)	
West	42 (4.77)	0.26 (0.17, 0.41)		0.26 (0.17,0.41)	
South	51 (4.20)	1.45 (0.09, 0.21)	<0.001	0.16 (0.11,0.25)	<0.001
Pregnancy characteristic					
Parity					
0	81 (3.31)	Ref		Ref	
1	389 (26.13)	2.61 (1.78, 3.85)		1.72 (1.09,2.71)	
≥2	1,031 (70.56)	3.98 (2.74, 5.75)	<0.001	1.87 (1.20,2.92)	0.02
Abortion characteristics					
Reason for getting abortion					
Therapeutic	99 (6.59)	Ref		Ref	
Humanitarian and social	1,351 (92.06)	6.45 (4.84, 8.59)		4.50 (3.34,6.07)	
Sex selective	15 (1.35)	2.33 (1.12, 4.85)	<0.001	1.56 (0.72,3.38)	<0.001

Table 5 assesses the factors that predisposed women to having unsafe abortions. Among the 5,856 women in the study sample who underwent an abortion, a total of 147 (2.43%) abortions were considered unsafe in this study. Almost 62% of the women who had unsafe abortions were in the 20-29 years age group, 20.77% of them were illiterate, and 72.71% resided in rural areas. A majority of women (57.72%) cited humanitarian or social reasons for undergoing an induced abortion. On adjusted analysis, women with higher educational levels were less likely (aOR=0.31 (0.14, 0.67)) to undergo an unsafe abortion compared to those with no education. Furthermore, women reporting humanitarian and social reasons for getting an abortion were less likely to undergo an unsafe abortion (aOR=0.54 (0.33, 0.87)) compared to those who cited therapeutic reasons. On the other hand, the women reporting sex-selective reasons had higher odds of engaging in an unsafe abortion (aOR= 1.61 (0.70, 3.70)). Moreover, no statistically significant association between parity and unsafe abortion was observed in the current study (aOR=0.76 (0.33, 1.73).

## Discussion

The findings of this largest nationally representative survey data from India indicate that nearly nine in 10 women in India utilize abortion care from private health facilities compared to public ones. Previous studies from India have reported that women felt discouraged to seek care from public facilities due to the requirement of repeated visits, longer waiting duration, and perception of poor preservation of confidentiality [13,28]. Compared to the previous round of the NFHS-4 (2015-16), the current round observed a similar proportion of women seeking abortion care at private rather than public healthcare facilities, regardless of their age, proximity to the facility, or financial status [29]. A multi-country comparative study observed that Bangladesh has better abortion care services at public health facilities compared to India and Nepal [30].

The present study observed a social gradient in the pattern of utilization of abortion care services with women belonging to lower social and economic classes undergoing abortions at home rather than visiting any health facility, a finding consistent with existing evidence from LMICs, suggesting that women having financial constraints probably avoid utilizing abortion services from healthcare facilities due to direct or indirect costs [28,31]. A previous study from Jharkhand, India, also previously observed that women who couldn't afford to pay for a qualified private physician sought care from unlicensed or government facilities [32]. A systematic review assessing knowledge, attitude, and practices of contraception and abortion among adolescents from LMICs inferred that the limited knowledge about legal laws regarding abortion in India and the prevailing stigma may encourage women to perform an abortion at home without the supervision of a registered health professional [33]. In this study, women who reported domestic violence were more likely to undergo an abortion at home but the findings lacked statistical significance. However, previously, underreporting of domestic violence due to the associated stigma and discrimination has been reported [34,35]. Consequently, prospective studies may be conducted to further evaluate this association. This study also found that low educational level is an independent risk factor for not utilizing abortion-care services through formal healthcare providers at designated health facilities instead of opting for self-managed abortions at home signifying the need to improve awareness of abortion-related laws and services in socioeconomically disadvantaged vulnerable populations.

This study corroborates the evidence from previous studies that indicate adolescent girls in LMICs tend to have poor abortion care-seeking behavior [32,36,37]. Stigma against single women, especially adolescents, precludes appropriate sexual health-seeking behavior including basic services such as contraceptive counseling [31,36,38]. Experiencing unintended pregnancies at an early age may detrimentally impact their education and employment opportunities that perpetuate emotional and financial distress. Moreover, adolescent girls tend to recognize and accept their pregnancies later, hence, they are more likely than comparatively older women to delay getting an abortion, which is associated with adverse physical and mental health agony [31].

The present study also noted regional variations in the utilization of public versus private health facilities among abortion care seekers. In the southern and western states of India, such as Kerala, Tamil Nadu, Andhra Pradesh, Telangana, Goa, and Maharashtra, a significant proportion of women underwent abortions at private facilities. Contrary to this, in the northeastern region of the country, the likelihood of getting an abortion at a private medical facility was manifold lower, which is consistent with prior evidence from India [23,39]. Factors that could explain this phenomenon include a higher unmet need for contraception, limited private abortion clinic infrastructure, reduced stigma related to abortion, and the lower population density in the Northeastern states of India [11].

The current study observed that apart from therapeutic reasons, such as medical complications in abortion, women reporting social and sex-selective reasons for abortion tend to avoid availing services at any healthcare facility. Previous evidence also indicated primary reasons for abortion in India are unwanted pregnancy and financial issues, which may also contribute to the burden of unsafe abortions [23,28]. Additionally, this study also suggests that women availing medication over surgical methods of abortion were significantly more likely to undergo an abortion at home without consultation of any formal healthcare provider. Such an attitude toward seeking abortion care in abeyance of medical consultation, especially when recommended after the first trimester of pregnancy may further accentuate the risk to the mother’s health [9].

Furthermore, in a previous study, it was observed that women reporting sex-selective reasons for having an abortion were the high-risk groups for engagement in unsafe abortion practices. India banned sex-determination way back in 1994, although, prenatal sex determination is still a prevailing concern in Indian society [40-42]. In other countries, such as China, Nepal, and Nigeria, induced abortions continue to be performed for sex-selective reasons [43-45].

Even though the MTP Act was amended in 2021 in response to numerous calls to improve access to safe abortions while safeguarding the confidentiality and autonomy of women, it only provides limited recognition of self-managed medication abortions [10]. Only 48% of induced abortions in rural areas were performed by doctors according to the previous NFHS fourth round (2015-16), and there was no change in this trend in the current NFHS fifth round (2019-21). However, there has been an increase in the proportion of induced abortions conducted by medical professionals in urban areas from 60% in 2015-16 to 66% in 2019-20. The remainder of the induced abortions were handled by nurses, assistant nurse midwives, dais (midwives), family members, or self. According to the Rural Health Statistics Report (2019-20), there exists a 69.7% shortage of obstetricians and gynecologists in community health centers in rural India compared to what was required for the available infrastructure with 56.1% vacant positions [46]. This lack of qualified medical personnel may continue to limit women's access to safe abortion procedures, particularly in rural India.

The major strengths of this study are the large, nationally representative data collection by trained enumerators. However, the study has certain limitations. The awareness of women regarding the abortion laws and their legal rights, which could have helped to understand the gaps in abortion-seeking practices, could not be assessed in this study as this information was not collected in this survey. Moreover, since women are often reluctant to report abortions due to stigma and discrimination, the number of abortions and possible reasons could be underreported in the dataset, especially those relative to illegal sex-selective abortions. Finally, most women in this sample were married while the occurrence of unsafe abortions is historically more likely among unmarried women resulting in a likely underestimation of the problem [47].

## Conclusions

Women in India, regardless of maternal age, empowerment status, or socioeconomic status, were more likely to utilize private health facilities compared to public ones for abortion care services. Self-managed abortions at home were more prevalent in women of lower socioeconomic status, adolescent girls, and in those reporting social and sex-selective reasons for abortion. The overall proportion of unsafe abortions was very low (2.5%), and most were attributed to humanitarian and social reasons, although higher educational levels were protective. Our study findings imply that improving awareness of legal rights and access to abortion care services through mass media campaigns, especially for women in rural India, may improve their utilization. Furthermore, the reproductive health program in India should enhance capacity-building initiatives for primary care providers including doctors, nurses, and pharmacists to effectively prescribe and supervise abortion through medication methods.

## Appendices

**Table 5:** Factors associated with the place of abortion (public, private, versus home) for abortion care utilization in women aged 15-49 years (unadjusted analysis) RRR: Relative risk ratio; SC: Scheduled Caste; ST: Scheduled Tribe; OBC: Other Backward Caste *Testparm p-value

Variables	Public Facility		Private Facility		
	RRR (unadjusted)	P-value	RRR (unadjusted)	P- value	Overall p-value*
Individual level					
Age					
≤19	Ref		Ref		
20-29	0.94 (0.46, 1.90)	0.85	0.71 (0.46, 1.90)	0.19	
30-39	1.31 (0.64, 2.70)	0.42	0.86 (0.51, 1.44)	0.57	
≥40	1.03 (0.35, 1.42)	0.94	0.69 (0.36, 1.29)	0.25	0.053
Marital status					
Unmarried	Ref				
Married	3.09 (0.99, 9.62)	0.052	0.79 (0.34, 1.82)	0.59	
Widowed/separated/divorced	4.58 (1.04, 20.19)	0.044	0.95 (0.29, 3.07)	0.93	0.17
Education attainment					
No education	Ref		Ref		
Primary	1.24 (0.86, 1.79)	0.24	1.37 (0.64, 1.20)	0.43	
Secondary	1.35 (1.02, 1.81)	0.03	1.37 (1.09, 1.72)	0.007	
Higher	1.45 (0.99, 2.13)	0.055	2.90 (2.14, 3.93)	<0.001	<0.001
Husband's education					
No education	Ref		Ref		
Primary	1.40 (0.30, 6.48)	0.66	0.53 (0.13, 2.16)	0.38	
Secondary	0.70 (0.24, 2.02)	0.51	0.59 (0.26, 1.35)	0.21	
Higher	0.63 (0.18, 2.15)	0.46	0.75 (0.28, 1.95)	0.54	0.63
Comprehensive women empowerment (N=911)					
No	Ref		Ref		
Yes	1.10 (0.65, 1.86)	0.71	1.67 (1.05, 2.64)	0.30	0.07
Exposure to mass media					
No exposure	Ref		Ref		
Any exposure	1.08 (0.85, 1.35)	0.53	1.67 (1.35, 2.04)	<0.001	<0.001
Domestic violence (N=753)					
No	Ref		Ref		
Yes	0.81 (0.48, 1.38)	0.45	0.71 (0.45, 1.14)	0.16	0.37
Correct knowledge of ovulatory cycle					
No	Ref		Ref		
Yes	1.16 (0.94, 1.44)	0.14	1.10 (0.92, 1.32)	0.28	0.32
Distance to health facility a problem					
No problem	Ref				
Big problem	0.98 (0.76, 1.26)	0.89	0.94 (0.76, 1.15)	0.53	
Not a big problem	1.09 (0.87, 1.36)	0.45	0.92 (0.76, 1.11)	0.39	0.59
Getting money for treatment					
No problem	Ref		Ref		
Big problem	1.21 (0.94, 1.56)	0.14	0.77 (0.62, 0.98)	0.03	
Not a big problem	0.95 (0.76, 1.19)	0.66	0.77 (0.64, 0.92)	0.005	<0.001
Household level					
Religion (N=5,821)					
Hindu	Ref		Ref		
Muslim	1.77 (1.29, 2.41)	<0.001	1.12 (0.85, 1.47)	0.40	
Other	2.24 (1.49, 3.35)	<0.001	1.79 (1.22, 2.61)	0.003	<0.001
Caste (N=5,507)					
SC	1.21 (0.89, 1.65)		0.77 (0.60, 0.99)		
ST	1.42 (1.00, 2.01)		0.58 (0.41, 0.83)		
OBC	1.04 (0.79, 1.37)	<0.001	0.88 (0.71, 1.10)	<0.001	<0.001
Non-SC/ST/OBC	Ref		Ref		
Household size					
<5 members	Ref		Ref		
5+ members	0.74 (0.61, 0.91)	0.003	0.86 (0.72, 1.01)	0.07	0.012
Wealth quintile					
Poorest	Ref		Ref		
Poorer	1.18 (0.89, 1.56)	0.25	1.27 (0.98, 1.65)	0.069	
Middle	1.43 (1.07, 1.92)	0.01	2.24 (1.71, 2.94)	<0.001	
Richer	1.18 (0.87, 1.60)	0.28	2.18 (1.66, 2.87)	<0.001	
Richest	0.89 (0.64, 1.24)	0.51	2.98 (2.25, 3.94)	<0.001	<0.001
Community level					
Place of residence					
Urban	Ref		Ref		
Rural	0.92 (0.73, 1.15)	0.46	0.67 (0.55, 0.81)	<0.001	<0.001
Region					
North	Ref		Ref		
Central	0.53 (0.39, 0.70)	<0.001	0.91 (0.71, 1.15)	0.43	
East	0.64 (0.47, 0.87)	0.005	0.71 (0.55, 0.91)	0.008	
Northeast	2.10 (1.55, 2.85)	<0.001	0.61 (0.45, 0.83)	0.002	
West	2.06 (1.28, 3.34)	0.003	4.94 (3.18, 7.65)	<0.001	
South	4.21 (2.83, 6.27)	<0.001	5.74 (4.02, 8.19)	<0.001	<0.001
Pregnancy characteristic					
Trimester					
First	Ref		Ref		
Second	0.98 (0.73, 1.32)	0.93	1.17 (0.92, 1.49)	0.18	
Third	1.10 (0.84, 1.44)	0.46	1.19 (0.95, 1.50)	0.12	0.43
Parity					
0	Ref				
1	0.50 (0.33, 0.76)	0.001	0.47 (0.33, 0.69)	<0.001	
>2	0.34 (0.23, 0.50)	<0.001	0.30 (0.21, 0.42)	<0.001	<0.001
Abortion characteristics					
Reason for getting abortion (N=5,519)					
Therapeutic	Ref		Ref		
Humanitarian and social	0.16 (0.12, 0.22)	<0.001	0.18 (0.14, 0.24)	<0.001	
Sex selective	0.27 (0.13, 0.57)	0.001	0.47 (0.24, 0.92)	0.30	<0.001
Method of abortion (N=5,619)					
Medicine	Ref		Ref		
Surgical	35.57 (22.79, 55.51)	<0.001	36.84 (24.00, 56.55)	<0.001	<0.001
Complications from abortion					
No	Ref		Ref		
Yes	1.17 (0.87, 1.56)	0.28	1.26 (0.99, 1.61)	0.057	0.16
